# Seroprevalence of infectious bursal disease in backyard chickens of selected districts of Buno Bedelle zone, Southwestern Ethiopia

**DOI:** 10.1002/vms3.1522

**Published:** 2024-07-25

**Authors:** Chali Deressa, Zelalem Abera, Geremew Batu

**Affiliations:** ^1^ Ayira District Livestock and Fisheries Development and Resource Office Ayira Oromiya Ethiopia; ^2^ Department of Veterinary Clinical Study, School of Veterinary Medicine Wallaga University Nekemte Ethiopia; ^3^ West Wallaga Zonal Livestock and Fisheries Development and Resource Office Gimbi Oromiya Ethiopia

**Keywords:** Bedelle, chickens, ELISA, Ethiopia, IBD, seroprevalence

## Abstract

**Background:**

Infectious bursal disease (IBD) is a viral infectious disease that affects young chicks. A cross‐sectional study was conducted from October 2020 to June 2021 to determine seroprevalence and associated risk factors of IBD on backyard chickens of purposively selected three districts of Buno Bedelle Zone, Southwestern Ethiopia.

**Methods:**

The study was carried out via a collection of serum samples, questionnaire surveys, and a total of 768 serum samples were collected from randomly selected backyard chickens of the study areas and examined for the presence of IBD virus. These serum samples were processed by using an indirect enzyme‐linked immunosorbent assay test procedure in laboratories. SPSS version 20 was used for data analysis and descriptive statics techniques were used.

**Results:**

Out of a total serum samples collected, 361 of them were found positive for the disease with an overall seroprevalence of 47%. Different seroprevalence rates of IBD with 55.9%, 43.3% and 41.2% were recorded in Didessa, Chora and Gechi districts, respectively. The result indicated that there was statistically significant difference (*p* < 0.05) of the disease among the three districts. Among the nine Peasant Associations (PAs) observed for seroprevalence of IBD, highest (62.9%) and lowest (34.20%) seroprevalence of the disease was recorded in Yembero and Shengela PAs, respectively. However, IBD was statistically significant in only Shengela PA of Chora district (*p* = 0.04, OR = 1.93 and 95% CI 1.04–3.58) and Seko PA of Gechi district (*p* = 0.05, OR = 1.79 and 95% CI 1.01–3.16). Even though sex, breed, age, source and management‐based seroprevalence was observed in the present study, the result indicated that there was only statistical significant difference (*p* = 0.004, OR = 0.62 and 95% CI 0.45–0.86) seen in which higher seroprevalence of IBD was determined in exotic (50.3%) than in local (38.6%) chickens breeds of the study areas.

**Conclusion:**

Generally, higher seroprevalence of IBD in the present study indicates a widely distribution of the disease and one of the potential threats for poultry production in the study areas. So, chicken vaccination and improved management system should be warranted in order to control the disease effectively.

## Graphical abstract

Infectious bursal disease (IBD) is a viral infectious disease that affects young chicks. A cross‐sectional study was conducted from October 2020 to June 2021 to determine seroprevalence and associated risk factors of IBD on backyard chickens of purposively selected three districts of Buno Bedelle Zone, Western Ethiopia. The study was carried out via a collection of serum samples, questionnaire surveys, and a total of 768 serum samples were collected from randomly selected backyard chickens of the study areas and examined for the presence IBD virus. These serum samples were processed by using an indirect enzyme‐linked immunosorbent assay (ELISA) test procedure in laboratories. SPSS version 20 was used for data analysis, and descriptive statics techniques were used. Out of a total serum samples collected, 361 of them were found positive for the disease with an overall seroprevalence of 47%.

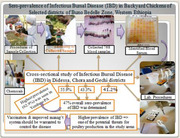

## 1 INTRODUCTION

Infectious bursal disease (IBD) is an acute, extremely contagious infection of young chickens (*Gallus gallus domesticus*). IBD or Gumboro disease is a highly contagious immunosuppressive viral infection of young chickens (3–6‐week old) causing severe economic and production losses (Muller et al., 2003). Ethiopia has the largest livestock population in Africa, with nearly 63 million cattle, over 31 million sheep and 33 million goats, and 61 million chickens in 2018. The sector contributed up to 40% of agricultural GDP, nearly 20% of total GDP and 20% of national foreign exchange earnings in 2017. Between 2000 and 2016, the average stock of livestock, measured in tropical livestock units (TLU) per 100 people, stood at 50.970 TLU, more than double the continental median of 23.44 TLU. At the same time, the average growth rate of gross production value during the same period was 4.54% that is also twice the continental median of 2.2% (Food and Agricultural Organizations [FAO]).

From the total population of chicken in Ethiopia, 99% are reared under the traditional backyard system of management, whereas 1% is under intensive management system. This flock provides with a yearly output of 72,300 metric tons of meat and 78,000 metric tons of eggs. Chickens are wide spread in the Ethiopia and are important to subsistence, economic and social livelihoods of a large human population. Chickens are especially important to women, children and aged individuals, who are the most vulnerable member of the society in terms of under‐nutrition and poverty (Central Statistics Authority [CSA], 2009; Teklewold et al., 2006).

There has been a gradual decline in the Ethiopian poultry population. According to the Central Statistical Authority (2004–2005), the Ethiopian poultry population was estimated at 85 and 31 million in 1954 and in 2005, respectively. The Sub‐Sector Review (1984) estimated the average number of chickens per household at 6.5 in 1984, whereas the average number of chickens per household is estimated at 4.1 in 2003. These figures show that the country's poultry population has turned down by 64% in the last 50 years, whereas the average number of chickens per farmer has reduced by 37% over the last 20 years. This problem attracts the attention of researchers in Ethiopia to improve health management and breeding aspect of chickens. In spite of the existence large population of chicken and potential future expansion of the poultry industry in the country, the production system has been adversely affected by a variety of constraints such as management problem (like nutrition, housing), predators and poultry diseases. Among the previous obstacles, the poultry diseases are the main constraints incriminated for the reduction of total numbers and compromised productivity (Ashenafi, 2000; CSA, 2013; Mazengia, 2012).

According to the work done by Ashenafi (2000), some farmers have stopped rearing chickens due to disease problems. Besides the above problem, imported breeds and cross‐breeds are multiplied in Ethiopia poultry hatchery centre, and day old chickens are imported from abroad. As a result, such new diseases are widely spreading throughout the country without being noticed and control measures in place with possible devastating effect on indigenous chickens. The occurrence of IBD was first reported in 2002 in Ethiopia at privately owned commercial poultry farm in which 45%–50% mortality rate was documented (Zeleke et al., 2005a).

Currently from those cited viral diseases, IBD is the most important threat to poultry production in the country (FAO, 1992; Solomon & Abebe, 2007; Zeleke et al., 2005b). Although the disease is one of the major health constraint viral diseases responsible for marked economic losses in a country, there is limitation of well‐documented information on the seroprevalence and associated risk factors of IBD, so far in the backyard chicken production system in the study areas. However, the reports of outbreak from the zone and questionnaire survey done on chicken mortality and assessment of chicken diseases carried out in the zone in 2017G.C by Beddelle Regional Veterinary Laboratory Center (BRVLC), there was no well organized data on ‘Sero‐prevalence on Infectious Bursal Disease in Backyard Chickens in selected districts of Buno Bedelle Zone’ to assess the prevalence of IBD and its associated risk factors in the study areas.

## 2 MATERIALS AND METHODS

### 2.1 Description of the study areas

A cross‐sectional study was conducted from October 2020 to June 2021 in apparently healthy backyard chickens (unvaccinated) that are found in the study areas. Three Kebeles (Peasant Associations [PAs]) were randomly selected from each district in consultation with the respective district, especially Livestock and Fisheries Development and Resource Office expert's. The zone is located 426 km far from Addis Ababa in the west direction. The zone was organized by nine districts and one town in March 2016. The zone covers 5856.5030 km^2^ of which 1126.64 km^2^ are covered by forests (CSA Results for Oromia Region, 2007).

Astronomically, this zone located at latitude and longitude lies between 8°27′ N and 36°21′ E. The zone contains highland (10%), midland (67%) and low land (23%) agro‐ecologies, and is located at an altitude ranging from 500 to 2575 m above sea level. The annual precipitation ranges from 1500 to 2200 mm with 6–9 months of rain fall. From the total human population of the zone is, about 88% of human populations reside in the rural areas. The farming system of the zone is characterized by mixed farming system, comprising both cropping and livestock production (Ministry of Agriculture [MOA], 2010).

#### 2.1.1 Didessa district

Didessa is one of the districts in Buno Bedelle Zone, which was named after Didessa River atributary of the Abay River. It is bordered on the south by Didessa River which separtes it from Jimma zone and on the North by Gechi. The major town is Didessa is Denbi. The total livestock population of the district are bovine: 95,513, goat: 19,460, sheep: 19,374, horse: 2808, mule: 2108, donkey: 2828 and poultry: 46,376. Among these livestock population of the district, bovine: 17,618, goat: 1937, sheep: 6680, horse: 1256, mule: 488, donkey: 1477 and poultry: 8799 were shared by Yembero, Sinesso and Gerado which are the selected PAs for the present study. Accordingly, bovine: 8589, goat: 611, sheep: 3114, horse:959, mule: 120, donkey: 417 and poultry: 3077; bovine: 4808, goat: 714, sheep: 1837, horse:224, mule: 134, donkey: 517 and poultry: 2253 and bovine: 8626, goat: 612, sheep: 1729, horse: 73, mule: 234, donkey: 543 and poultry: 3469 were found in Yembero, Sinesso and Gerado which were the selected PAs of the district for the present study, respectively (Profile of Buno Bedele Zone Livestock Resource Office [PBBLRO]; CSA Results for Oromia Region, 2007).

#### 2.1.2 Chora DIt is one pofistrict

It is one of the districts of Buno Bedelle zone. It is bordered on the south by Jimma zone on the west by Yayo on the northwest by Supena Sodo, on the north by Dega and on the east by Bedelle. The major town in Chora is KumbabeThe total livestock population of the district is bovine: 172,235, goat: 42,640, sheep: 76,082, horse: 10,320, mule: 2820, donkey: 2560 and poultry: 168,517. Among these livestock population of the district, bovine: 20,072, goat: 5021, sheep: 6324, horse: 460, mule: 305, donkey: 1523 and poultry: 13,267 were shared by Shengela, Hawa Yember and Aba Bora which are the selected PAs of Chora district. Accordingly, bovine: 3702, goat: 767, sheep: 1593, horse: 62, mule: 9, donkey: 41 and poultry: 4394; bovine: 2830, goat: 654, sheep: 411, horse: 48, mule: 146, donkey: 1477 and poultry: 2235 and bovine: 13,540, goat: 3600, sheep: 4320, horse: 350, mule: 150, donkey: 5 and poultry: 6638 were shared by Shengela, Hawa Yember and Aba Bora, respectively (CSA Results for Oromia Region, 2007; PBBLRO).

#### 2.1.3 Gechi district

**FIGURE 1:**
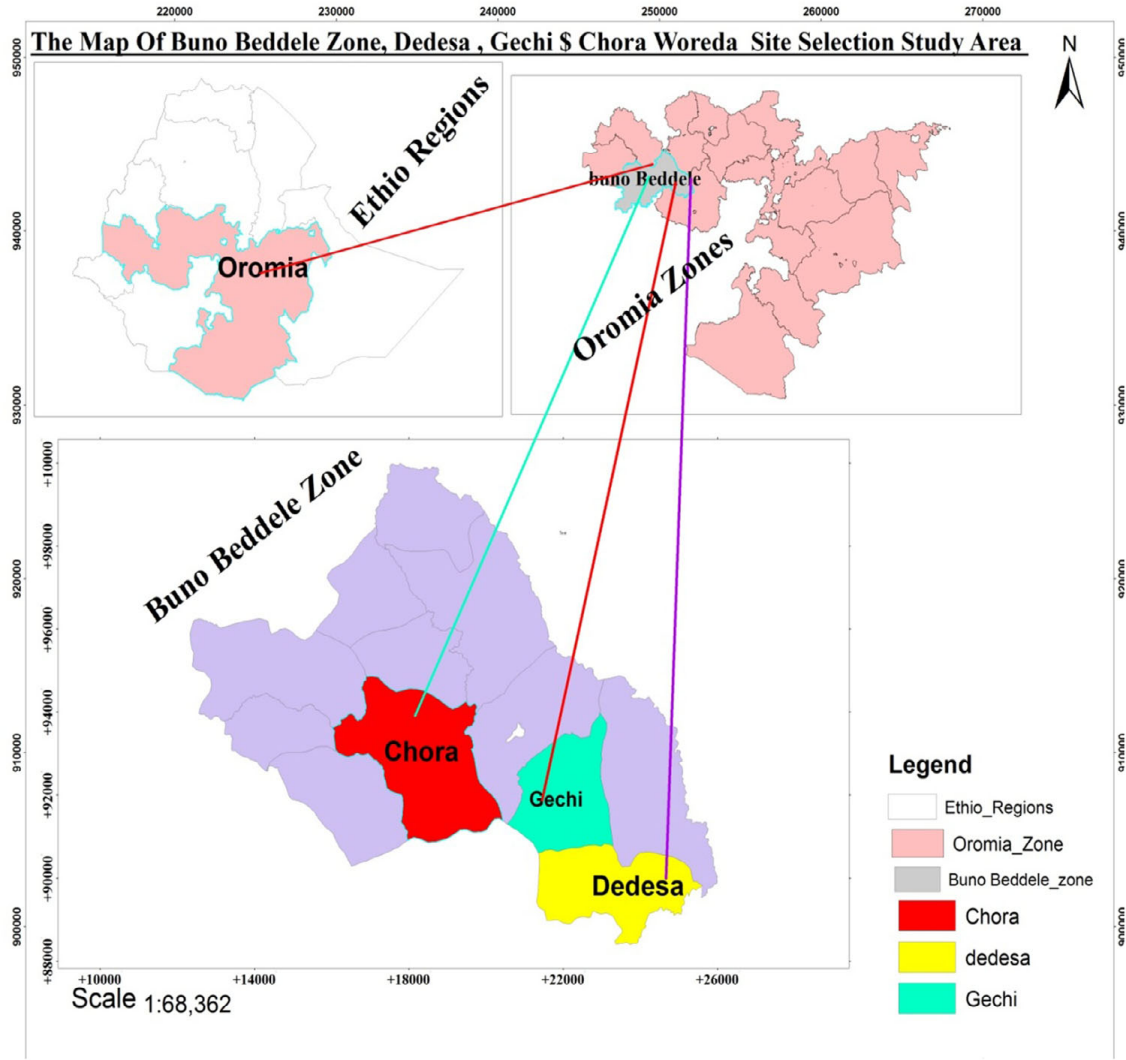
Maps of the study areas.

It is one of the districts of Buno Bedelle zone in the Oromia regional state, Ethiopia. The district is bordered on the south by Didessa district, on the north by Bedelle district and in the east by the Didessa river which separates it from Jimma zone. The major town is Gechi. The total livestock populations of the district are bovine: 1393,521, goat: 31,852, sheep: 35,571, horse: 9702, mule: 1154, donkey: 6367 and poultry: 120,488. Among these livestock population of the district, bovine: 21,512, goat: 4529, sheep: 6058, horse: 875, mule: 61, donkey: 352 and poultry: 12,530 are shared by Gole Maya, Seko and Gechi 01 Kebeles of the Gechi district. Accordingly, bovine: 7985, goat: 1200, sheep: 815, horse: 180, mule: 28, donkey: 300 and poultry: 4350; bovine: 2768, goat: 249, sheep: 326, horse: 160, mule: 9, donkey: 8 and poultry: 2590; and bovine: 10,759, goat: 3080, sheep: 4917, horse: 535, mule: 24, donkey: 44 and poultry: 5590 livestock populations were found in Gole Maya, Seko and Gechi 01 which were the selected PAs of the district, respectively (PBBLRO; CSA Results for Oromia Region, 2007) (Figure 1).

### 2.2 Study population

In this study, age, breed sex and origin were taken as potential risk factors. The age of the chicken was classified as adults (>12 months) and young (6–12 months). The age was determined subjectively based on the size of crown, length of spur and flexibility of the xiphoid cartilage according to Magwisha et al. (2002) together with information from the poultry farmers. Chickens were classified as their origin of the selected districts. Based on the sex, chickens were also grouped as male and female. From the total 768 study animals, the blood samples were randomly taken from 263 (34.2%), 228 (29.7%) and 277 (36.10%) chickens from Didessa, Chora and Gechi district, respectively, based on the variation of chicken population in the districts during the study period.

### 2.3 Study design

A cross‐sectional study was conducted from November 2020 up to June 2021 to determine the seroprevalence and its associated risk factors in the study area. The simple random sampling technique was followed to select the chickens from households. A semi‐structured questionnaire interview of selected household owner was used to assess factors associated with the occurrence of IBD virus (IBDV). Age, sex, breed, flock size, hygiene condition (good and poor), based on real current status of backyard chickens (hygiene condition of study area), source of chicken (home, purchased), district, housing system (separated house, roost in family dwelling or kitchen) and the presence or the absence of exotic breed among flock were emphasized as risk factors (Appendices I).

### 2.4 Sample size determination

The sample size was determined according to Pfeiffer (2002) and Thrusfield (2007). As there was no prior similar research work conducted in the study area, expected animal level prevalence of 50% was assumed to get the maximum number of sample size required. The absolute precisions were decided to be 5% at 95% confidence level. Accordingly, a sample size of 384 was obtained using the following formula:

$$n=\frac{1.96^{2}P_{\exp}\left(1-P_{\exp}\right)}{d^{2}}$$

where *n* is the required sample size, *P*_exp_ is the expected prevalence, and *d* is the desired level of precision (5%), through the Office of International des Epizooties (OIE) recommended diagnostic tool.

Therefore,

$$n=\frac{{\left(1.96\right)}^{2}\times 0.5\times \left(1-0.5\right)}{0.0025}=\frac{3.8416\times 0.25}{{\left(0.05\right)}^{2}}=384$$

However, based on the calculation of the above formula, the sample size was doubled to increase the precision of the study; accordingly 768 samples were collected.

### 2.5 Sampling technique and sample collection methods

The blood samples were collected from apparently healthy and unvaccinated backyard chickens from the three purposively selected districts. About 2–3 mL of blood was aseptically collected from the branchial (wings) vein of apparently healthy chickens using 5 mL sterile disposable syringe with 22 ga and 11/4 needle size. The whole blood collected from the chickens were labelled and allowed to clot for 3–4 h at 4°C, then the syringes were placed horizontally at 45° to allow sera separation. The separated serum was transferred into each labelled sterile Cryovials tube and then kept cool for transportation to the laboratory. The sera in the tube were stored at −20°C until being tested. Then each serum sample was subjected to the laboratory test.

The three study area districts were purposively selected based on chickens diseases outbreak reports and a questionnaire survey done on backyard chick mortality in 2017 by BRVLC and proposed from districts of the zone. Then, we identified chickens population, the number of PAs, of districts, road accessibility and others with district and Zone Livestock Resource and Development Office professionals. After discussing in detail with zone and district professionals, we selected three PAs from each district randomly by lottery method. Then before starting our work, we had discussed about the study with PAs managers, Livestock and Fisheries Development and Resource Office expert's and Developmental Agency professionals, and we collected necessary information like number of PAs households, chickens population, how they made awareness creation to farmers, objectives of the study and others. As described in Table 1, the numbers of chickens population and PAs were different to make the number of samples per household proportional; 1, 2, 3, 4, 5, 6, 7, 8,9,10 and 12 samples of flocks 2–3, 4, 5–6, 7–8, 9–10, 11–12, 13–15, 16–17, 18–19, 20–22 and above 23 samples were taken, respectively.

**TABLE 1:** Population of chickens and number of households (HHs) per Peasant Association (PAs).

District	PAs	Number of HHs/PA	Number of chickens/PAs	Number of selected HHs	Number of samples taken per PAs
Didessa	Yembero	704	176	16	89
	Sinesso	756	189	21	95
	Garado	650	154	21	79
Chora	Shengela	620	154	22	79
	Hawa Yember	568	142	30	70
	Aba Bora	628	157	20	79
Gechi	Gole Maya	670	155	25	79
	Seko	830	207	22	106
	Gechi 01	720	180	23	92
Total		6112	1514	200	768

*Source*: PBBLRO.

So samples were collected from above 6‐month age group of randomly selected apparently health chickens of randomly selected households per PAs. Number of samples per district, PAs and household were determined depending on their respective number of chickens population and were collected proportionally.

### 2.6 Laboratory diagnostic methods

#### 2.6.1 ELISA test procedure, validity and interpretation

Indirect enzyme‐linked immunosorbent assay (ELISA) was performed in the laboratory, on all sera samples collected according to OIE (2004). The procedure employed in the laboratory was used an ID Screen IBD Indirect – ID.vet innovative diagnostic indirect ELISA (Louis Pasteur‐Grabels, France) kit which is used to detect the presence of anti‐IBD antibodies in the chicken serum following the kit manufacturers’ recommended protocol. The test sera were pre‐diluted by dilution buffer 14 in a pre‐dilution plate according to the established protocol or kit instructions, and each was dispensed into the requested number of micro wells. In the ELISA plate pre‐diluted samples and dilution buffer 14 were added and incubated for 30 ± 3 min at 21°C. After incubation, the sera were discarded from the plates, and each well was washed three times by 300 µL of washing solution. About 100 µL anti‐chicken horseradish peroxidase conjugate was dispensed into the wells, and the plates were incubated for 30 ± 3 min at 21°C. After incubation, again the sera were discarded from the plates, and each well was washed three times by 300 µL of washing solution. About 100 µL substrate solutions were dispensed into each test well and again incubated for 15 ± 2 min at 21°C in the dark place. After a final incubation, the substrate solution (TMB) was stopped by adding about 100 µL stop solution, and the colour reactions were quantified by measuring the optical density (OD) of each well at 450 nm.

To check the validity of IBD ELISA result, validity test was done. In valid IBD ELISA result, the mean OD value of positive control serum is greater than 0.250, and the ratio of the mean value of the positive and negative control is greater than 3. For the interpretation of the result, serum sample positive (SP) control ratio was required. If SP value was >0.3, the IBD antibody status was considered to be positive, but ≤0.3 was taken as negative (Annex IV).

For each sample, calculate the S/P ratio and antibody titre as follows:

Sample positive (S/P) ratio:

$$S/P=\frac{\mathrm{OD}\mathrm{sample}-\mathrm{ODNC}}{\mathrm{ODPC}-\mathrm{ODNC}}$$

Antibody titre:

$$\begin{array}{c} \mathrm{Log}10\left(\mathrm{titre}\right)=0.97\times \mathrm{Log}10\left(S/P\right)+3.449\\ \mathrm{Titre}=10\;\mathrm{Log}10\left(\mathrm{titre}\right)\;\left(\mathrm{Annex}\mathrm{IV}\right). \end{array}$$

### 2.7 Data analysis

The data collected and results for the seroprevalence were entered to MS excel spread sheet programme to create data base, which was filtered before analysed by using SPSS version 20 (SPSS, 2011). The chi‐square test was used for the association of OD and risk factors. The association between seroprevalence rate and explanatory factors (age, sex and management status, source of chickens and place of origin) was carried out by way of chi‐square (*χ* ^2^) test. Significant difference was held at 95% and *p*‐values were less than 0.05 in all analyses. Seroprevalence was determined using the total number of positive sera samples divided by the total number of sera tested. For analysis of serological data, the chickens were divided into two groups: those with ELISA OD value ≤0.3 and the others with >0.3 (protective against IBDV).

### 2.8 Data quality control and quality assurance

The quality of the data was assured via a careful development of the sample collection format and questionnaire format for data collection after a thorough literature review. The questionnaire was prepared first in English and translated into Afan Oromo language (the local language) and retranslated into English to check its consistency. The questionnaire format was validated using a pre‐test on 5% of the sample size that was randomly selected, and appropriate modifications were made (Annex II).

## 3 RESULTS

### 3.1 Overall seroprevalence of infectious bursal disease (IBD) in backyard chicken

Out of 768 total sera samples collected from backyard chickens of the districts and tested using indirect ELISA test, 361 of them were infected with an overall seroprevalence of 47% recorded in the study areas (Table 2).

**TABLE 2:** Summary of infectious bursal disease (IBD) seroprevalence by different variables.

Variables	Category level	No. of examined	No. of positive	Prevalence (%)	*p*‐Value	OR	95% CI	
							Lower	Upper
Districts	Didessa	263	147	55.9	0.004	0.60	0.43	0.85
	Chora	228	94	41.2	0.64	1.09	0.76	1.55
	Gechi	277	120	43.3	–	–	–	–
Sex	Female	654	306	46.8	0.77	1.06	0.71	1.58
	Male	114	55	48.3				
Age	6–12 months	454	216	47.6	0.70	0.95	0.71	1.26
	>12 months	314	145	46.2				
Breed	Exotic	553	278	50.3	0.004	0.62	0.45	0.86
	Local	215	83	38.6				
Management system	Semi‐intensive	107	45	42.1	0.27	0.79	0.52	1.20
	Backyard	661	316	47.8				
Source of chicken	Purchased	464	215	46.3	0.65	0.93	0.70	1.25
	Hatched at home	304	146	48.0				
Total		768	361	47				

### 3.2 Seroprevalence of IBD with respect to age, sex, management system, source and area

#### 3.2.1 District level seroprevalence

The study comprised district, PAs, sex, age, management and source as major factors that play a role for the infection of IBD in the study areas in which seroprevalence of the disease in chickens were recorded as 55.9%, 41.2% and 43.3% in Didessa, Chora and Gechi district, respectively. Therefore, the result indicated that there was statistically significant difference (*p* < 0.05) in Didessa district among the three districts.

#### 3.2.2 Sex‐wise seroprevalence

On the other hand, an association between the disease and sexes of animals (female and male) was studied and out of examined chickens, the majority or 654 (85.20%) of them were females, whereas about 114 (14.80%) of them were males. The result indicates that higher female chickens were sampled to observe the seroprevalence of IBD in sex level of chickens in which 48.20% male and 46.8% female chickens were positive for the disease. This shows that a relatively higher seroprevalence rate was recorded in males than in females (Table 2).

#### 3.2.3 Age‐wise seroprevalence

In the present study, the seroprevalence of IBD was 47.6% in young chickens (6–12 months) and 46.2% in adult chickens with more than 12 months of ages, in which slightly higher prevalence of the disease (47.6%) was observed in young than in adult chickens (46.20%) (Table 2).

### 3.3 Breed‐wise seroprevalence

During this study, an effort was also made to observe seroprevalence of IBD among chicken breeds. From a total of 215 local and 553 exotic breeds of chickens examined for the presence of IBD, 38.6% and 50.3% local and exotic breeds were found infected with IBD, respectively. This indicates that higher seroprevalence of IBD was determined in exotic than in local breeds of chickens and there was statistically significant difference (*p* < 0.05) of the disease occurrence between breeds of the chickens (Table 2).

### 3.4 Management‐wise seroprevalence

An effort was also made to observe management wise seroprevalence of IBD in which majority or 86.1% of them were from backyard (free range), whereas 13.9% of the chickens were from semi‐intensive farming system. Out of the examined chickens from both management types, 47.80% and 42.05% chickens were tested positive for the disease in backyard (free range) and semi‐intensive farming system, respectively. The result shows that slightly higher seroprevalence was determined in chickens from backyard (free range) farming system (Table 2).

### 3.5 Source‐wise seroprevalence

Analysis of source‐wise seroprevalence of IBD was also conducted in which 48% and 46.3% of them were from hatched and purchased chickens, respectively. The result of analysis indicates that relatively higher seroprevalence was observed in hatched than purchased chickens. However, statistical analysis of result indicated that there was no statistically significant difference (*p* > 0.05) seen in seroprevalence of IBD among management, source, age and sex groups of chickens in the study areas (Table 2).

### 3.6 Seroprevalence of IBD at the level of Peasant Associations (PAs)

Different seroprevalence rates of IBD were observed among nine PAs of the three districts with 62.9%, 49.5%, 55.69%, 34.20%, 35.70%, 53.20%, 45.56%, 35.85%, 50% from Yembero, Sinesso and Gerado PAs of Didessa district; Shengela, Hawa Yember and Aba Bora PAs of Chora district; Gole Maya, Seko and Gechi 01 PAs of Gechi district, respectively. The study revealed that highest (62.9%) and lowest (34.20%) seroprevalences of the disease were recorded in Yembero PA and Shengela PA, respectively. However, among the nine PAs of the three districts, seroprevalence of IBD was statistically significant (*p* < 0.05) in only Shengela and Seko PAs of Chora and Gechi district, respectively (Table 3).

**TABLE 3:** Seroprevalence of infectious bursal disease (IBD) at the level of Peasant Associations (PAs).

Selected districts	PAs	No. of examined	No. of positive	Prevalence (%)	*p*‐Value	OR	95% CI	
							Lower	Upper
Didessa	Yembero	89	56	62.6	0.08	0.59	0.33	1.07
	Sinesso	95	47	49.5	0.94	1.02	0.58	1.81
	Gerado	79	44	55.7	0.46	0.79	0.44	1.45
Chora	Shengela	79	27	34.2	0.04	1.93	1.04	3.58
	Hawa Yember	70	25	35.7	0.07	1.80	0.95	3.40
	Aba Bora	79	42	53.2	0.68	0.88	0.48	1.61
Gechi	Gole Maya	79	36	45.6	0.56	1.19	0.65	2.18
	Seko	106	38	35.8	0.05	1.79	1.01	3.16
	Gechi 01	92	46	50.0	‐	‐	‐	‐
Total		768	361	47				

## 4 DISCUSSIONS

In the present study, out of 768 total sera samples collected from backyard chickens of the districts and tested using indirect ELISA test, 361 of them were positive for IBD with an overall seroprevalence of 47%, indicating IBD is wide spread and statistically significant in the study areas (OR = 0.60, CI: 0.43–0.85, *p* = 0.004) (Table 2). A number of sero‐diagnostic tests are available for the detection of the serum antibodies against IBD (Hussain et al., 2004). ELISA has been reported to be very sensitive in the diagnosis of IBD in chickens (Amin et al., 1999). IBD is one of the diseases that cause chick mortality (Olwande, 2014). There has been increasing interest to estimate the prevalence of IBD antibody in indigenous free‐range chickens as 40%–60% of the chicks hatched die during the first 8 weeks of life (Central Agricultural Census Commission, 2003), mainly due to disease and predation.

According to this study, the overall seroprevalence of IBD in the study area was slightly higher than the study done around Mekelle town, Northern Ethiopia 45.05% by Zegeye et al. (2015). The result of the study was in agreement with the report of Lemma et al. (2019) who reported 51.7% in non‐vaccinated chickens of Jijiga and Harar districts and Mazengia et al. (2008) who reported an overall prevalence of 51.1% from Bahir Dar and Farta districts on back yard chicken production systems.

In contrasting to the result of the present study (47%), far higher seroprevalence was reported by Nigussie (2007), which is 61% in backyard chickens of Addis Ababa and Adami Tulu areas; Kassa and Molla (2012) reported 75% in North Gondar and West Gojjam of northern Ethiopia; Swai et al. (2011) reported 82.5% in northern Tanzania; Zeryehun and Fekadu (2012) reported 82% in central Oromia; Degefa et al. (2012) reported 76.6% in western shewa of Oromia regional states of Ethiopia; Reta (2008) reported 76.3% of Zeleke et al. (2005b) who reported 93.3%; and Woldemariam and Wossene (2007) reported 100% in the non‐vaccinated backyard chickens using I‐ELISA test. This prevalence was also lower than the studies of Lawal et al. (2014), Jenbreie et al. (2013) and Hailu et al. (2010) with the prevalence of 63.5%, 83.1%, 76.64%, respectively, in different parts of Ethiopia in indigenous free‐range chickens.

Individual chicken level sero‐prevalence of IBD in this study was higher, as compared to the reports of Reta (2008) in East Shoa Zone (39.2%) using Agar gel immuno‐diffusion test; Durojaiye and Kwenkam (1990) in indigenous village chickens in Cameroon (33.9%); Anjum et al. (2003) in backyard chickens in Pakistan (34%); Mazengia et al. (2010) in Ethiopia (29.4%); Hailu et al. (2009) in Ethiopia (38.4%); and Shettima et al. (2018) in Nigeria (33.4%).

The present study conducted on backyard chickens revealed that different seroprevalence rates of the disease were recorded in chickens as 55.9%, 41.2% and 43.3% in Didessa, Chora and Gechi districts, respectively. Therefore, the seroprevalence of IBD was statistically significant (*p* < 0.05) among chickens sampled from Didessa districts as compared with those chickens sampled from Chora and Gechi districts. This relatively higher prevalence of the disease in Didessa district may be associated with the larger poultry farms in Denbi town of Didessa district than other districts. This might also be due to the IBDV that is very resistant to different environmental conditions and is capable of surviving in the environment for long period.

The result of the present study obtained in PAs of each selected districts indicates that 62.9%, 49.5%, 55.69% were from Yembero, Sinesso and Gerado PAs of Didessa district, 34.2%, 35.7% and 53.2% were from Shengela, Hawa Yember and Aba Bora PAs of Chora district, and 45.56%, 35.85% and 50% were from Gole Maya, Seko and Gechi 01 PAs of Gechi district, respectively (Table 3). This PAs level seroprevalence was almost related with the overall seroprevalence (47%) of the study area. This might be due to the similarity of the chicken's management practices and the presence of more related chickens flock sizes of the farmers in their respective PAs. This is also in agreement with Van Den Berg (2000) in the nature of the disease, as there is no specific environmental situation that can prevent or modify the occurrence of the disease.

In this study, slightly higher seroprevalence of IBD (48.25%) was recorded in male than in female chickens (46.80%). However, result of analysis using binary logistic regression indicates that sex was not significantly associated with the occurrence of IBD (OR = 1.06, CI: 0.71–1.58, *p* = 0.77) (Table 2). This might be due to the fact that both males and females can be equally infected by IBDV. This finding in agreement with previous studies reported by Bedaso et al. (2017), Degefa et al. (2012), Jenbreie et al. (2013), Kassa and Molla (2012), Swai et al. (2011), Zeryehun and Fekadu (2012) showed that there was no significant association of seroprevalence among sexes. Reta (2008) also reported the absence of influence of sex on the prevalence of the disease. In contrast to the current study, reports from Bettridge et al. (2014) and Zegeye et al. (2015) showed that seroprevalence difference between males and females could be due to physiological and immunological difference between the two sexes.

The age range of chickens used for this study was included only the chickens above 6 months, so as to exclude chickens with maternally derived antibody. Because maternal antibodies to IBD in unvaccinated chickens persist in chicks up to 21 days as determined by ELISA with complete decay by 28 and 35 days (Zaheer & Saeed, 2003). With the exclusion of chickens with maternally derived antibody and vaccination history, the antibody detected in the chickens would have been caused by a field virus, as the chickens were on free range. This implies that the field virus is capable of inducing a higher antibody titre level between the prevalence of both age groups (Table 2).

Accordingly, seroprevalence of the present study indicates that 47.57% and 46.20% was from 6 to 12 months (young) and chickens greater than 12 months (adult) age groups, respectively. Binary logistic regression analysis (OR = 0.95, CI: 0.71–1.26, *p* = 0.70) shows no statistical difference between both age groups. This might be due to an equal exposure of both age groups and constant re‐infection with the field virus during scavenging their feed. The disease occurs worldwide in all major poultry production areas and it can be serologically evident in all age groups, and IBDV is very resistant to different environmental condition, which is capable of surviving in the environment for long period (Van Den Berg, 2000).

IBD is highly contagious disease and it is described as if one chicken is found positive in a certain flock, the whole flock is considered infected (OIE, 2008). In this study, exotic backyard chickens and Horro local backyard chickens were compared and a higher seroprevalence IBDV antibody rate of 50.3% than Horro local backyard chickens (38.60%) were found at individual level of chickens, the difference of which was statistically significant (*p* < 0.05) (Table 2). This might be due to the reason that exotic breed chickens have the ability to scavenging from contaminated materials than local chickens. So, exotic backyard chicken breeds were compared to local backyard Horro breeds. The current study is in‐line with Natnael (2015) who described the lowest seroprevalence IBD antibody in local backyard breeds (48.31%) compared to exotic breeds (67.11%) in and around Bahir Dar, North West Ethiopia. This indicated that the indigenous breed of chickens was also exposed to IBDV and this agrees with the study of Mazengia et al. (2009) in Bahir Dar and Farta and also (Zeryehun & Fekadu, 2012) in central Oromia.

In the present study, chickens managed under semi‐intensive farming system are also included because these chickens are partially managed under extensive farming system. Accordingly, the seroprevalence of IBD in free range farming system (backyard) is higher (47.8%) than that of semi‐intensive farming system (42.05%), but the difference was not statistically significant (OR = 0.79, CI: 0.52–1.20, *p* = 0.27) (Table 2). This might be because both free‐range and semi‐intensive chickens were in contact with different contaminated materials and infected chickens on the field and the sanitary status of both free‐range and semi‐intensive chickens roosting place might be the same.

In the present study, the seroprevalence of IBD was 48% and 46.3% from chickens hatched at home and purchased from the market, respectively. But result of analysis using binary logistic regression indicates that source or origin of chicken was not significantly associated with the occurrence of IBD (OR = 0.93, CI: 0.70–1.25, *p* = 0.65) (Table 2). This might be because chickens from both sources were living in the same environment and there was no quarantine measures practised to limit the spread of the virus between both chicken groups. The ease of contact at local open‐air markets between chickens from different areas, which are then taken back to various localities, can undoubtedly facilitate the rapid spread and persistence of IBD among backyard chickens. Some husbandry practices may favour the spread and maintenance of this economically important infectious disease (Sule et al., 2013), including: inappropriate sanitary conditions, nutritional deficiencies, continuous exposure to wild birds, absence of routine vaccination, rearing of different species of birds together and mixing of chicken during transit and at points‐of‐sale in markets (Swai et al., 2011). Many of these factors were observed in the study area, for example chicken freely scavenging and mixing with other chicken breeds from the neighbours while searching for feed, and returning chickens from the markets. These activities readily facilitated the transmission of IBDV in backyard chicken. The IBDV can survive for long in the environment, thus enhancing its transmissibility (Mutinda et al., 2014).

## 5 CONCLUSSION AND RECOMMENDATIONS

IBD or Gumboro disease is an acute, extremely contagious infection of young chickens which is the most important threat to poultry production. The study was conducted in apparently healthy backyard chickens (unvaccinated) that are found in Didessa, Chora and Gechi districts of Buno Bedelle Zone. The study comprised districts, PAs, sex, breeds, age, source and management to determine seroprevalence and associated risk factors of IBD on backyard chickens. Out of the total serum samples collected and examined using Indirect ELISA test, 361 of them were tested positive for IBDV antibody with an overall seroprevalence of 47%. The result reveals that there is a high seroprevalence of IBD in the study area, which could seriously affect the rearing of chickens in the backyard production system. The disease was found to have a higher seroprevalence in Didessa than Chora and Gechi districts, which requires a serious attention. Semi‐structured questionnaire was designed to collect information from 200 households of the study areas using a face‐to‐face interview which involves the contents such as socio‐demographic data of respondents, flock size, management, history of the disease occurrence, health care and constraints and prevention, control and farmer's awareness. So, advising famers to get their chickens vaccinated is a necessary step in reducing the prevalence besides maintaining hygienic condition of environment in which the chickens are rearing, Having immunization schedules with active follow‐up in backyard chickens is crucial. Furthermore, continuous surveillance should be implemented for a better understanding of the epidemiology of the disease.

## AUTHOR CONTRIBUTIONS

Chali Deressa has made significant contributions to conception and design, acquisition of data, analysis and interpretation of data and writing – original draft. Zelalem Abera has made important contributions to conception and design, supervision, analysis and interpretation of data and revising the manuscript critically for important intellectual content. Geremew Batu has been involved in conception and design, acquisition of data, supervision and revising the manuscript.

## CONFLICT OF INTEREST STATEMENT

The authors declare no conflicts of interest.

## ETHICS STATEMENT

Submitted by the certificate number of Vet.Med/ERC/13/2020

### PEER REVIEW

The peer review history for this article is available at https://publons.com/publon/10.1002/vms3.1522.

## Data Availability

The data collected and used to support this article can be offered by the first or corresponding author upon request.
